# Prevention and control strategies for children Kashin–Beck disease in China: A systematic review and meta-analysis

**DOI:** 10.1097/MD.0000000000016823

**Published:** 2019-09-06

**Authors:** Fang-fang Yu, Xin Qi, Yan-na Shang, Zhi-guang Ping, Xiong Guo

**Affiliations:** a Department of Epidemiology and Biostatistics, College of Public Health, Zhengzhou University, Zhengzhou; b NHC Key Laboratory of Trace Elements and Endemic Diseases, Institute of Endemic Diseases, School of Public Health of Health Science Center, Xi’an Jiaotong University, Xi’an, China.

**Keywords:** Kashin–Beck disease, meta-analysis, prevention strategy

## Abstract

**Background:**

To evaluate prevention and control strategies for children with Kashin–Beck disease (KBD) in China through a systematic review and meta-analysis.

**Methods:**

We conducted literature searches of articles indexed in Web of Knowledge, PubMed, Springerlink, Elsevier, the Chinese National Knowledge Infrastructure, and Wanfang data until February 2019. Search terms included “Kashin–Beck disease” or “KBD,” and “improvement of water” or “change of grain” or “salt-rich selenium” or “comprehensive measures.” Eligible studies were prospective trials of interventions in endemic area. Data extraction was performed by 2 independent authors using predefined data fields that also included quality evaluation.

**Results:**

We screened 1183 potentially relevant articles, and included 22 studies that reported 24 trials, with data from 3700 healthy children and 2961 children KBD. The pooled odds ratios (ORs) and confidence intervals (95% CIs) for primary prevention new incidence in healthy children following interventions to comprehensive measures, change of grain, salt-rich selenium, and improvements of water were 0.15 (0.02, 0.95), 0.15 (0.03, 0.70), 0.19 (0.09, 0.38), and 0.20 (0.09, 0.42), respectively. The OR (95% CI) for clinical improvement in children KBD following interventions to improvement of water, salt-rich selenium, comprehensive measures, and change of grain were 5.03 (3.21, 7.89), 4.39 (3.15, 6.11), 2.98 (1.61, 5.52), and 2.35 (1.59, 3.47), respectively. All interventions showed significant differences and were effective (*P* < .05).

**Conclusion:**

Comprehensive measures and change of grain were the most effective measures in preventing new case, whereas improvement of water and salt-rich selenium resulted in clinical improvements in children KBD.

## Abstract 2

Supplemental Digital Content is available in the text

## 1 Introduction

Kashin–Beck disease (KBD) is an endemic and chronic osteochondropathy involving with growth plate and articular cartilage. Its geographical distribution ranging from Southeastern Siberia to China in Asia.^[1,2]^ Its clinical manifestations included joint pain, enlarged and deformed joints, mobility disorders, and limb muscle atrophy, severe cases present as dwarfism and disability.^[3,4]^ KBD is prevalent in 14 provinces (352 counties) in China, with an incidence of approximately 820,000 cases, and mainly distributed in the remote western China (Shannxi, Sichuan, Gansu, Qinghai provinces, as well as Tibet).^[1]^ The X-ray detection rate of KBD has been estimated at 11% with a positive rate of 8% to 15%.^[5]^ Identifying effective strategies for preventing and treating KBD requires further research.

Over the past 160 years, more than 50 potential environmental risk factors have been investigated by researchers, who identified several environmental factors that can trigger related auxiliary factors, and result into KBD. At present, the KBD is considered to be induced by humic acid in drinking water, cereal crop contamination by mycotoxin-producing fungi, and selenium deficiency.^[4]^ However, none of these hypothesis satisfactorily explain the pathogenesis of KBD, suggesting a complex network of biologic and environmental factors. Public health organizations in China have implemented large-scale prevention and control measures in endemic areas, to control and reduce the new incidence of KBD. As a result, the overall trend of KBD has been decreased with some success.^[5]^ But the incidence is still considerable in western China, the Ganzi and Aba regions of Sichuan and the Changdu region of Tibet.

We systematically evaluated and compared KBD prevention measures from the perspective of the 3 major environmental etiology hypotheses, to elucidate the causes of the decline in new cases. Effect prevention and control strategies will assist in manage the incidence of children KBD in China.

## 2 Methods

### 2.1 Search strategy

We conducted a literature search of all publications indexed until February 2019 using Web of Knowledge, PubMed, Springerlink, Elsevier, the Chinese National Knowledge Infrastructure, and Wanfang data. The following terms were searched: “Kashin–Beck disease” or “KBD” and “improvement of water” or “change of grain” or “salt-rich selenium” or “comprehensive measures” in both English and Chinese. Additional studies were identified by contacting subject-matter experts and by performing manual searches of unpublished materials.

### 2.2 Study selection

Studies were included if they met the following criteria: Study design: A community based trial with different intervention measures. Study subjects: patients with KBD were confirmed diagnosis by the diagnostic criteria for KBD and subjects who resided in the endemic areas. Intervention measures: improvement of water from deep wells and house tap, change of grain was replaced with the wheat, flour, and rice from the non-KBD areas, salt-rich selenium with a salt/selenium ratio of 1:60,000, and comprehensive measures (approaches combining all above 3 measures). Primary outcomes: new cases of KBD in healthy children and clinical improvement (metaphysis and epiphysis repair) in children KBD identified by X-ray films. Studies were excluded based on the following: comparative data were not available between 2 groups, we have not access to the full text of the study through various channels, and or other interventions were also carried out.

To determine the eligibility for inclusion, 2 investigators (FFY, XQ) independently scanned titles and abstracts. A study was excluded when it met exclusion criteria. Disagreements were resolved by discussion with authors (ZGP, XG).

### 2.3 Data extraction

The following information was extracted and entered into a predefined datasheet by 3 investigators (FFY, YNS, and ZGP): general information including title, author names, publication date, and data source; study characteristics including demographics, study area, study duration, baseline comparability, study objectives, and results; and outcome measures such as new incidence, and clinical improvement (metaphysis and epiphysis repair). If relevant information regarding design or outcomes was unavailable, or there was doubt regarding duplicate publications, the original authors were contacted for clarification. Differences were resolved by discussion with authors (ZGP and XG).

### 2.4 Methodologic quality assessment

The methodologic quality of the studies was assessed using a checklist described by Higgins et al,^[6]^ and covered the generation of groups, blinding, ascertainment, follow-up, comparability, and analysis. Disagreements were resolved through discussion.

### 2.5 Analytical strategy

To assess prevention and control strategies, we used the odds ratio (OR) for new incidence and clinical improvement attributable to the interventions. The studies assessed 2 cohorts: healthy children in the endemic area who received interventions to prevent new cases of KBD, and children KBD in the endemic area who received interventions and showed clinical improvements (metaphysis and epiphysis repair). This study was a systematic review and meta-analysis, and ethical approval and informed consent were not sought.

A database with the abstracted data was created using Stata version 14.0 (Stata Corporation, College Station, TX). We used the metan command in Stata to estimate the impact of each study (OR and 95% CI), random effects or fixed effect summary estimates, and heterogeneity statistics. Estimates of heterogeneity were obtained using the Mantel–Haenszel model. Summary estimates, 95% CIs, and weights of each study were estimated using the DerSimonian and Laird random effects model. Heterogeneity was determined by the Chi-squared test with significance set at *P* < .1. *I*^2^ was used to estimate total variation between studies, and *I*^2^ > 50% was considered substantial heterogeneity. Sensitivity analyses were conducted to evaluate the robustness of the pooled results, and all analyses were repeated for studies with large (>100) and small (≤100) samples. Funnel plots and Begg test were used to assess publication bias. Two-tailed tests were used for all statistical analyses, and *P* < .05 was considered statistically significant.

## 3 Results

**Figure 1:**
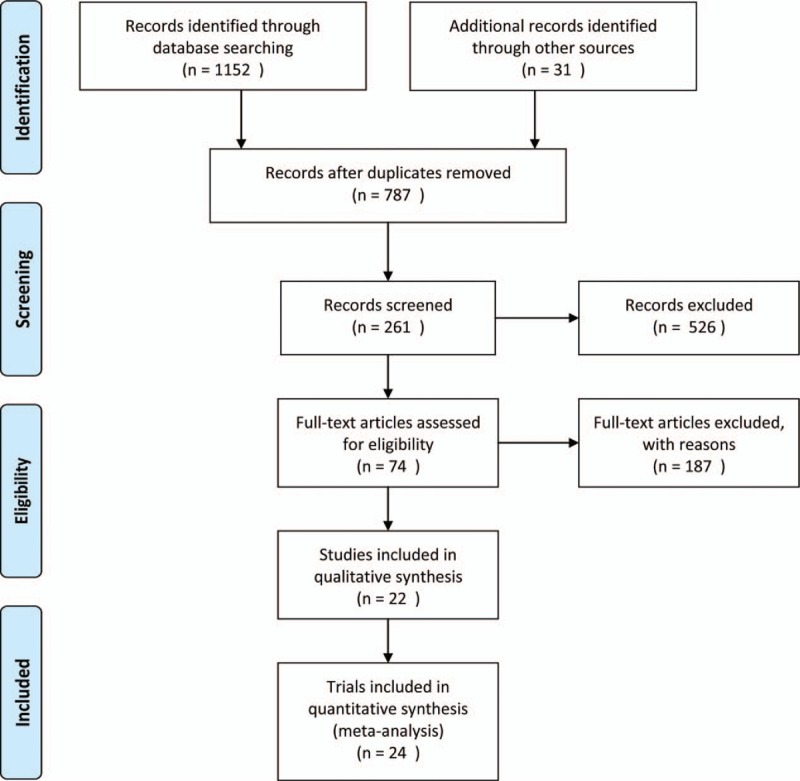
Flow diagram of included study.

We identified 1183 records, from which 74 full-text articles were selected for eligibility. Of these, 10 studies reported improvement of water, 14 studies reported change of grain, 38 studies reported salt-rich selenium, and 12 studies used comprehensive measures. Fifty-three articles did not to meet the inclusion criteria and were excluded. Four studies did not provide controls, comparative data, or newspaper coverage. Finally, we identified 22 studies that included 24 trials (Fig. 1). Five studies (6 trials) reported improvements of water,^[7–11]^ 3 studies (4 trials) reported change of grain,^[12–14]^ 11 studies (11 trials) reported salt-rich selenium,^[15–25]^ and 3 studies (3 trials) reported comprehensive measures.^[26–28]^

### 3.1 Baseline characteristics

Table 1 shows the identified 22 studies (24 trials), which involved 6661 participants (3700 healthy children and 2961 children KBD in endemic areas). Sample sizes in interventions and controls were as follows: improvement of water (n = 6, 880 interventions and 559 controls), change of grain (n = 4, 879 interventions and 572 controls), salt-rich selenium (n = 11, 1677 interventions and 975 controls), and comprehensive measures (n = 3, 264 interventions and 855 controls). All subjects were under the age of 20 years, and follow-up was 10 to 96 months. The effect indicator for prevention and control was: no new cases in healthy children and clinical improvement in children KBD.

**Table 1:**
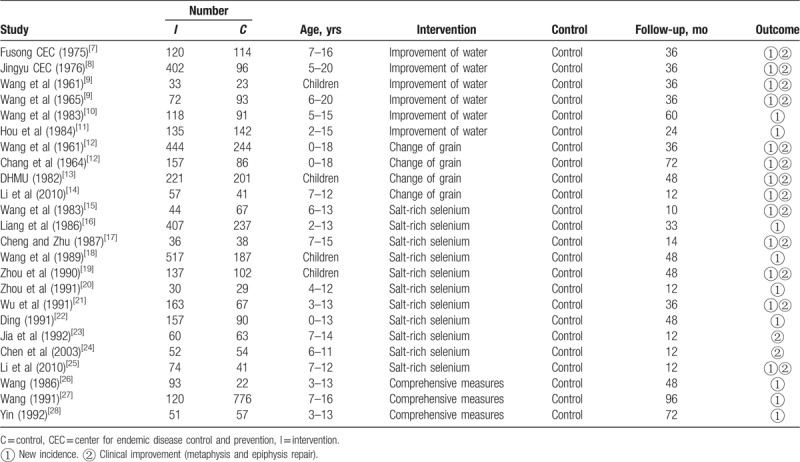
Baseline characteristics.

### 3.2 Evaluation quality of community-based trial

The methodologic quality of the 24 trials was summarized in Table 2. All trials implemented different interventions in the village and a similar characteristics neighboring village was used as a control, and 16 trials have compared geography, diet, nature, customs, water, soil, grain, and incidence at baseline. Allocation concealment and blinding method were not clear or mentioned by any of them. All trials reported the equal follow-up periods for cases and groups, and 12 trials have had completeness of follow-up, duration of follow-up varied from 10 to 96 months. No intention-to-treat analysis has been presentation in the 24 trials. Additionally, some samples were small and may have contributed to biases in the studies associated with these trials.

**Table 2:**
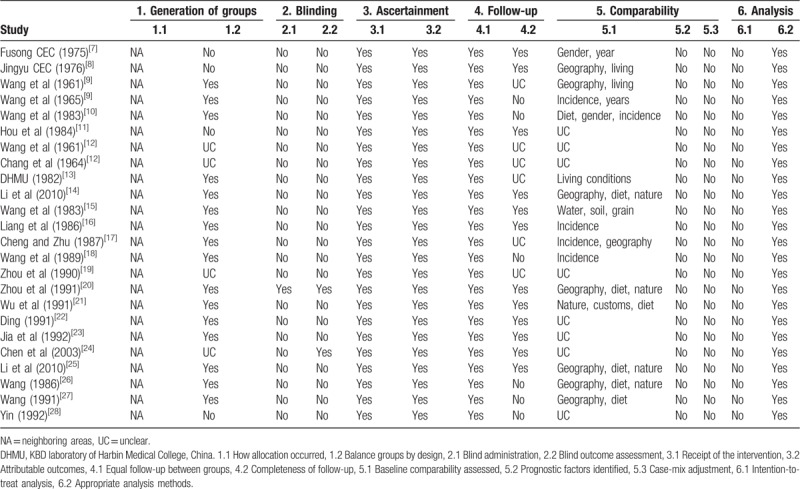
Methodologic quality of community-based trial.

### 3.3 Meta-analysis of interventions for prevention KBD in healthy children

**Figure 2:**
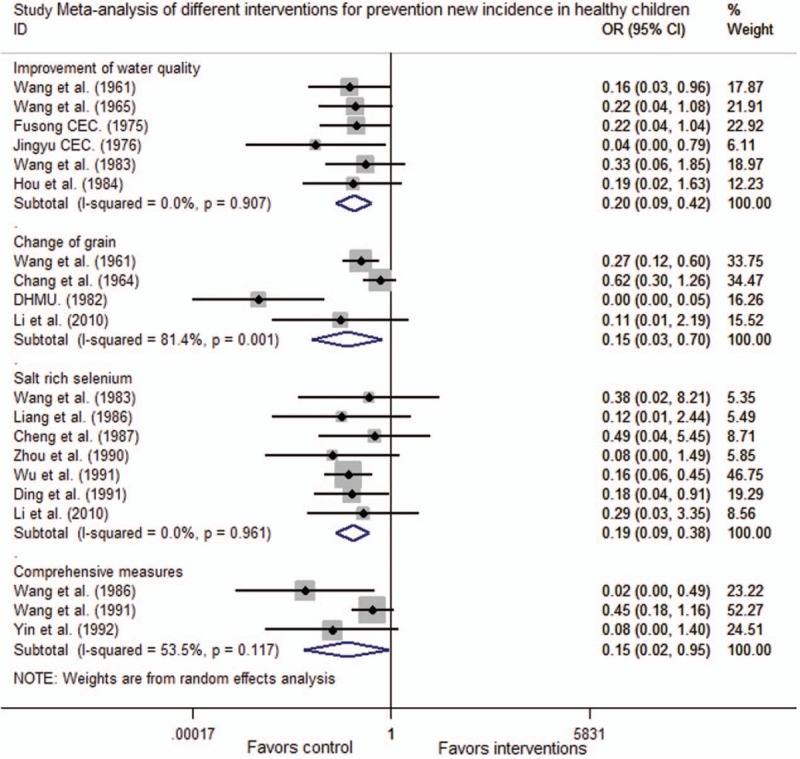
Meta-analysis of different interventions for prevention new incidence in healthy children. CI = confidence interval, OR = odds ratio.

Six trials assessed changes following an intervention to improvement of water. No heterogeneity was observed, and the pooled OR (95% CI) was 0.20 (0.09, 0.42), indicating the efficacy of improvement of water (*Z* = 4.24, *P* < .001). Four trials assessed changes following an intervention to change of grain, these trials had high heterogeneity and a pooled OR (95% CI) of 0.15 (0.03, 0.70), indicating the efficacy of change of grain (*Z* = 2.41, *P* = .016). Seven trials assessed changes following an intervention to salt-rich selenium, these trials had no heterogeneity and a pooled OR (95% CI) of 0.19 (0.09, 0.38), indicating the efficacy of salt-rich selenium (*Z* = 4.63, *P* < .001). Three trials assessed the efficacy of comprehensive measures, these trials had moderate heterogeneity, and a pooled OR (95% CI) of 0.15 (0.02, 0.95), indicating the efficacy of comprehensive measures (*Z* = 2.01, *P* = .044). From these findings, we determined that change of grain and comprehensive measures were the most effective strategies, to prevent new incidence of KBD in healthy children (Fig. 2).

### 3.4 Meta-analysis of interventions for clinical improvements in children KBD

**Figure 3:**
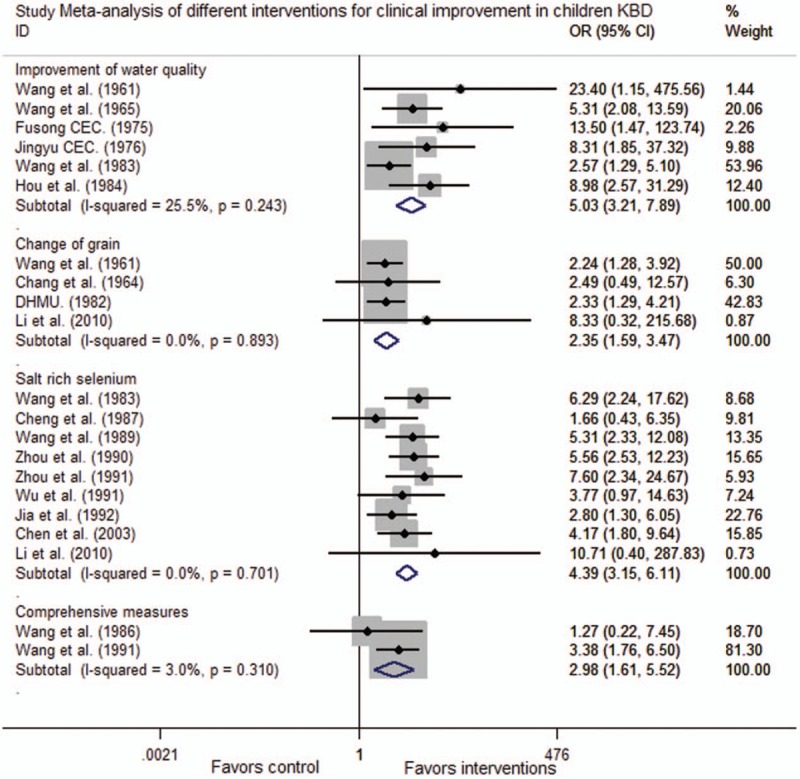
Meta-analysis of different interventions for clinical improvement in children Kashin–Beck disease. CI = confidence interval, OR = odds ratio.

Six trials assessed changes following an intervention to improvement of water. No heterogeneity was observed, and the pooled OR (95% CI) was 5.03 (3.21, 7.89), indicating the efficacy of improvement of water (*Z* = 7.03, *P* < .001). Four trials assessed changes following an intervention to change of grain. No heterogeneity was observed, and the pooled OR (95% CI) was 2.35 (1.59, 3.47), indicating the efficacy of change of grain (*Z* = 4.28, *P* < .001). Nine trials assessed changes following an intervention to salt-rich selenium. No heterogeneity was observed, and the pooled OR (95% CI) was 4.39 (3.15, 6.11), indicating the efficacy of salt-rich selenium (*Z* = 8.77, *P* < .001). Two trials assessed the efficacy of comprehensive measures. No heterogeneity was observed, and the pooled OR (95% CI) was 2.98 (1.61, 5.52) indicating the efficacy of comprehensive measures (*Z* = 3.48, *P* < .001). Clinical improvements in children KBD were seen in trials that improvement of water and salt-rich selenium (Fig. 3).

### 3.5 Sensitivity analyses

**Figure 4:**
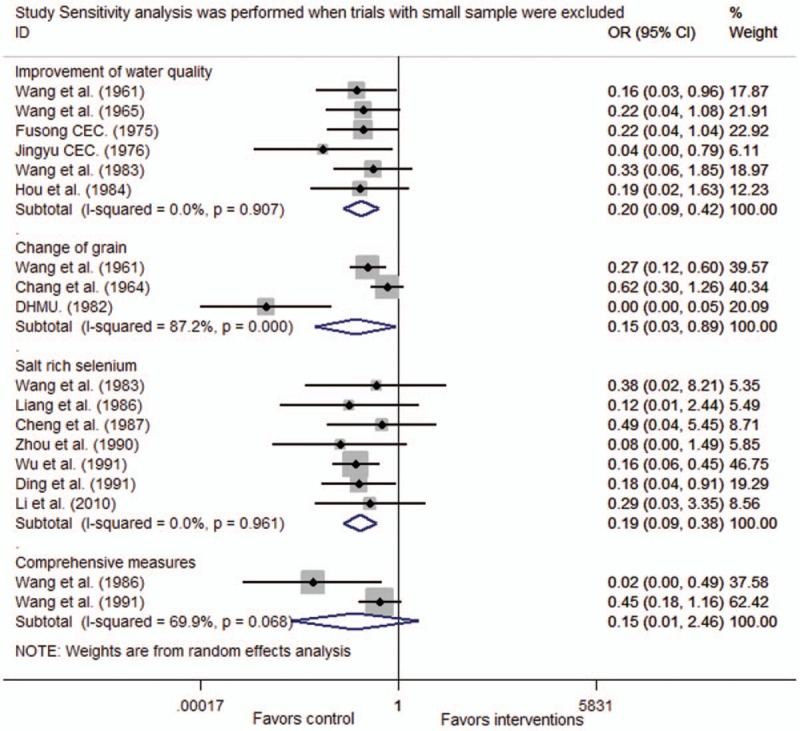
Sensitivity analysis were performed when trials with small sample were excluded. CI = confidence interval, OR = odds ratio.

For prevention new incidence in healthy children, heterogeneity was observed in trials that change of grain and comprehensive measures. The pooled OR did not change when we excluded trials with fewer than 100 participants^[14,28]^ reported comprehensive measures were excluded considering the sample size less than 100, and the sensitivity analyses were consistent with the pooled OR calculations (Fig. 4).

### 3.6 Publication bias

Begg test showed that there was no marked asymmetry in the funnel plots on preventing new incidence in healthy children for interventions that improvement of water (*Z* = 1.50, *P* = .13), change of grain (*Z* = 0.34, *P* = .73), salt-rich selenium (*Z* = 0.30, *P* = .76), and comprehensive measures (*Z* = 1.04, *P* = .30) (Supplementary Figures 1–8). There was no marked asymmetry in the funnel plots on clinical improvements in children KBD for interventions that improvement of water (*Z* = 1.50, *P* = .13), change of grain (*Z* = 1.70, *P* = .09), salt-rich selenium (*Z* = 0.10, *P* = .92), and comprehensive measures (*Z* = −1.00, *P* = .32). These findings indicated that the results have not publication bias.

## 4 Discussion

The KBD is painful and exert a heavy burden on society and their families. Effective intervention measures are necessary to prevent and control KBD in children at risk. Many epidemiologic investigations confirmed that KBD has a complex etiology closely associated with factors such as water, soil, and diet.

### 4.1 Humic acid in drinking water and KBD

The association of KBD with drinking water had been established as early as 1908, and early proposed interventions included boiling contaminated water prior to consumption. In the 1960s, KBD was suggested to be associated with organic plant matter in drinking water,^[29,30]^ which was reported to be acidic in endemic areas and to contain higher concentrations of fulvic acid than water in the nonendemic areas. Animal experiments have shown that fulvic acid can damage bone and cartilage tissue, induce deformation and fibrosis of chondrocytes in the knee joint.^[31]^ Higher levels of humic acid in drinking water, the result of pollution by organic substances from plant remains, have also been suggested to be involved in the pathogenesis of KBD.

This meta-analysis found out administration of improvement of water from well and tap water in an endemic village, a neighboring village with similar living conditions was used as control. The pooled OR (95% CI) for primary prevention in healthy children was 0.20 (0.09, 0.42) and for clinical improvement of children KBD was 5.03 (3.21, 7.89). Based on these findings, improvements of water can be effective in the prevention and control KBD. However, this hypothesis has its limitations: KBD has not been shown a dose–response relationship with humic acid levels in drinking water, and there were no significant differences in humic acid levels between endemic and nonendemic areas^[32]^; The animal models for KBD could not be reproduced using the humic acid of water in endemic areas.^[33]^

### 4.2 Contaminated grains and KBD

Toxins and metabolites produced by *Fusarium graminearum* have been suggested as a cause of KBD. Sergievsky and Rubinstein^[34]^ proposed that the incidence of KBD in the Soviet Union between 1932 and 1945 was attributable to grain contaminated by *F oxysporum* and its toxins, which cause cartilage damage. In the 1995, Yang had investigated the edible grain in the seriously endemic areas, as well as the grain in nonendemic areas, the results showed that the concentration of T-2 toxin in the homemade flour of KBD family has a positive rate of 67%, which was significantly higher than the commercial flour in nonendemic areas.^[33]^ Therefore, T-2 toxin concentrations in homemade flour may also cause KBD in China.

This meta-analysis found out administration of change of grain in an endemic village, the “change of grain” included 2 alternatives: ocal grain was exchanged by the wheat or flour from the non-KBD areas or national grain, and local grain was replaced with the rice from the non-KBD areas or national grain. A similar neighboring village was considered as a control. The pooled OR (95% CI) for primary prevention in healthy children was 0.15 (0.03, 0.70), and was 2.35 (1.59, 3.47) for clinical improvement in children KBD. Therefore, change of grain is an effective prevent and control measure for KBD. However, this hypothesis remains has its limitations. It is hard to explain the epidemiology focal distribution of KBD in terms of the different temperature, humidity, grain harvest, and storage conditions. Mycotoxin contamination by T-2 toxin was also widespread in non-KBD areas. Strains isolated from each endemic region were varied, and it was difficult to determine whether the toxins were endemic or nonendemic. Ten new cases of children KBD showed that 11 types of *Fusarium* toxins in serum were no positive.^[35]^*Fusarium* toxins can induce various types of tissue and cell damage without specificity toxicity on chondrocyte.^[36]^

### 4.3 Selenium deficiency and KBD

A biogeochemical hypothesis suggests that lack of certain chemical elements and compounds may affect mineral metabolism in the body and induce KBD. Vinogradov from the Soviet Union first put forward that many trace elements in soil, water, and crops were imbalance in the endemic areas. In the 1970s, Chinese researchers found out that low selenium levels have been closely associated with KBD and gradually developed into selenium deficiency hypothesis. Two meta-analyses concluded that sodium selenite was more effective in preventing and treating KBD.^[37,38]^

This meta-analysis found out administration of salt-rich selenium in the village, and used a similar neighboring village as control. The pooled OR (95% CI) for primary prevention in healthy children was 0.19 (0.09, 0.38), and the pooled OR for clinical improvement in children KBD was 4.39 (3.15, 6.11). Therefore, salt-rich selenium can be effective in the prevention and control measure for KBD. However, this measure remains has its limitations. KBD may not occur in low selenium areas, whereas KBD also may occur in normal selenium levels counties.^[39]^ Prospective studies had not shown a dose–response relationship between low selenium and KBD.^[40]^ Laboratory animals fed low-selenium diets did not develop necrosis of the epiphyseal plate and articular cartilage, a hallmark of KBD.^[41]^

Each of these etiologic hypotheses was insufficient by itself to elucidate the pathogenesis of KBD, suggesting that KBD develops from a combination of environmental risk factors. We determined that consuming grain from areas without KBD is an effective prevention strategy in healthy children, while improving the quality of drinking water is beneficial for patients with KBD, and enriching salt with selenium may be protective. Combining all 3 strategies is an effective measure of prevention and control.

## Author contributions

**Conceptualization:** Fang-fang Yu, Xin Qi, Yan-na Shang, Xiong Guo.

**Data curation:** Fang-fang Yu, Xin Qi, Yan-na Shang, Xiong Guo.

**Formal analysis:** Fang-fang Yu.

**Funding acquisition:** Xiong Guo.

**Writing – original draft:** Fang-fang Yu, Xin Qi, Zhi-guang Ping, Xiong Guo.

**Writing – review & editing:** Fang-fang Yu, Yan-na Shang, Zhi-guang Ping, Xiong Guo.

## Supplementary Material

Supplemental Digital Content
