# Supplementary material for: Prevention and control strategies for children Kashin–Beck disease in China: A systematic review and meta-analysis
Source: Medicine (Baltimore). 2019 Sep 6;98(36):e16823. doi: 10.1097/MD.0000000000016823 (PMC6738986; doi:10.1097/MD.0000000000016823)
Supplement: Supplemental Digital Content [file medi-98-e16823-s001.docx]

**eFigure1. Funnel plot of improvement of water for prevention new incidence in healthy children**

**eFigure2. Funnel plot of change of grain for prevention new incidence in healthy children**

**eFigure3. Funnel plot of salt rich selenium for prevention new incidence in healthy children**

**eFigure4. Funnel plot of comprehensive measures for prevention new incidence in healthy children**

**eFigure5. Funnel plot of improvement of water for clinical improvement in children KBD**

**eFigure6. Funnel plot of change of grain for clinical improvement in children KBD**

**eFigure7. Funnel plot of salt rich selenium for clinical improvement in children KBD**

**eFigure8. Funnel plot of comprehensive measures for clinical improvement in children KBD**
